# Along for the ride or missing it altogether: exploring the host specificity and diversity of haemogregarines in the Canary Islands

**DOI:** 10.1186/s13071-018-2760-5

**Published:** 2018-03-19

**Authors:** Beatriz Tomé, Ana Pereira, Fátima Jorge, Miguel A. Carretero, D. James Harris, Ana Perera

**Affiliations:** 1 CIBIO, InBIO - Research Network in Biodiversity and Evolutionary Biology, Universidade do Porto, Campus de Vairão, Rua Padre Armando Quintas, 4485-661 Vairão, Portugal; 2 Departamento de Biologia, Faculdade de Ciências da Universidade do Porto, Rua do Campo Alegre, 4169-007 Porto, Portugal; 3 Department of Zoology, University of Otago, 340 Great King Street, PO Box 56, Dunedin, 9054 New Zealand

**Keywords:** Apicomplexa, Hemoparasites, Lizards, *Gallotia*, *Tarentola*, *Chalcides*, Insularity, Phylogeography, *18S* rRNA

## Abstract

**Background:**

Host-parasite relationships are expected to be strongly shaped by host specificity, a crucial factor in parasite adaptability and diversification. Because whole host communities have to be considered to assess host specificity, oceanic islands are ideal study systems given their simplified biotic assemblages. Previous studies on insular parasites suggest host range broadening during colonization. Here, we investigate the association between one parasite group (haemogregarines) and multiple sympatric hosts (of three lizard genera: *Gallotia*, *Chalcides* and *Tarentola*) in the Canary Islands. Given haemogregarine characteristics and insular conditions, we hypothesized low host specificity and/or occurrence of host-switching events.

**Methods:**

A total of 825 samples were collected from the three host taxa inhabiting the seven main islands of the Canarian Archipelago, including locations where the different lizards occurred in sympatry. Blood slides were screened to assess prevalence and parasitaemia, while parasite genetic diversity and phylogenetic relationships were inferred from *18S* rRNA gene sequences.

**Results:**

Infection levels and diversity of haplotypes varied geographically and across host groups. Infections were found in all species of *Gallotia* across the seven islands, in *Tarentola* from Tenerife, La Gomera and La Palma, and in *Chalcides* from Tenerife, La Gomera and El Hierro. *Gallotia* lizards presented the highest parasite prevalence, parasitaemia and diversity (seven haplotypes), while the other two host groups (*Chalcides* and *Tarentola*) harbored one haplotype each, with low prevalence and parasitaemia levels, and very restricted geographical ranges. Host-sharing of the same haemogregarine haplotype was only detected twice, but these rare instances likely represent occasional cross-infections.

**Conclusions:**

Our results suggest that: (i) Canarian haemogregarine haplotypes are highly host-specific, which might have restricted parasite host expansion; (ii) haemogregarines most probably reached the Canary Islands in three colonization events with each host genus; and (iii) the high number of parasite haplotypes infecting *Gallotia* hosts and their restricted geographical distribution suggest co-diversification. These findings contrast with our expectations derived from results on other insular parasites, highlighting how host specificity depends on parasite characteristics and evolutionary history.

**Electronic supplementary material:**

The online version of this article (10.1186/s13071-018-2760-5) contains supplementary material, which is available to authorized users.

## Background

Understanding what determines the ability of a parasite to colonize and remain associated with a host is central to parasite evolution [1–4]. One of the most decisive factors shaping parasite distribution is host specificity [5, 6]. This trait determines the range of hosts a parasite can successfully infect and its ability to establish in new environments. The degree of host specificity is influenced by two main filters: ‘encounter’ probability with potential hosts, and ‘compatibility’, i.e. if the host provides suitable resources and the parasite can overcome host defenses [7–9]. Moreover, host specificity is not fixed in time, and plays a key role in parasite diversification, for example through co-speciation or expansion to novel hosts [4, 10, 11]. As such, the range of hosts a parasite can infect depends on ecological, physiological, geographical and historical factors [5, 8], alongside parasite transmission dynamics and life-cycle complexity [12–15]. To study host specificity, ideally the whole community of potential hosts of a parasite has to be considered. In this context, oceanic islands present a naturally simplified version of ecological interactions, being characterized by a depauperate and dis-harmonic fauna with high levels of endemics, which resulted from the non-random arrival of a small subset of the mainland pool followed by diversification processes [16, 17]. Colonizers face different selective pressures, which promote loss of genetic variability, increase in densities, niche broadening, among others [18]. These features are collectively referred to as the Island Syndrome. In the case of parasites, studies suggest that in insular systems they exhibit reduced diversity, higher prevalence values and an enlargement of the ecological niche, which is reflected in more frequent host-switching and more generalist parasites, in comparison to the mainland [19, 20].

Haemogregarines (Apicomplexa, Coccidia, Adeleorina) are intraerythrocytic parasites that infect a wide variety of vertebrates [21]. They have a heteroxenous life-cycle involving a hematophagous invertebrate vector (definitive host) and one vertebrate host (intermediate host) [22]. Additionally, these parasites can be transmitted *via* predation between vertebrate hosts [23–25]. Haemogregarines include three families: Haemogregarinidae, Karyolysidae and Hepatozoidae [21]. According to several phylogenetic studies, the family Haemogregarinidae (represented by the genus *Haemogregarina*) forms a separate clade, while the other families might not be monophyletic [26]. The genera *Hemolivia* and *Karyolysus* (both from the family Karyolysidae) clustered separately inside the genus *Hepatozoon* (family Hepatozoidae), leading some authors to propose a rearrangement of their taxonomy [27]. Moreover, a growing number of studies have uncovered high levels of undescribed diversity within this group ([26] and references therein), further complicating the taxonomical status of these parasites. Haemogregarines have generally been regarded as having low host specificity toward their vertebrate hosts [22, 28, 29]. However, patterns remain partially obscure as a combination of different factors complicate this assessment, including local host availability and distribution, transmission dynamics, co-evolutionary history and taxonomical uncertainties [23, 29–31].

**Fig. 1:**
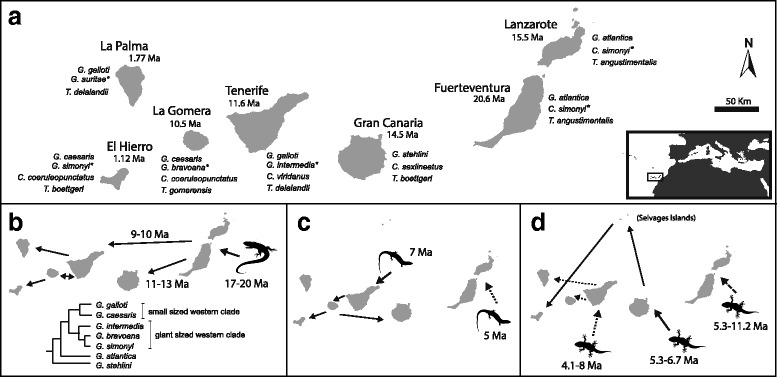
Study area and host lizard colonization history. **a** Map of the Canary Islands. Beside each main island are the island emergence age and the lizard species that inhabit it (of the genus *Gallotia*, *Chalcides* and *Tarentola*, endangered species are indicated by an asterisk). **b** Estimated time and colonization routes for the genus *Gallotia*, with the phylogenetic relationships between species (according to Cox et al. [52]). **c** Estimated time and colonization routes for the genus *Chalcides* [53]. **d** Estimated time and colonization routes for the genus *Tarentola* [54]

The Canary Islands have been the setting of several parasitological works (e.g. [32–34]), some of them reporting cryptic diversity in cestodes and nematodes [35, 36]. In a recent study on intestinal nematodes of the genus *Spauligodon*, Jorge et al. [37] found four lineages infecting Canarian lizards, three of which present host-switching events dating to the time of parasites’ colonization. However, this widening of host breadth seems to have been temporary, as contemporary host-parasite associations show a high level of host specificity, differing from the Island Syndrome expectations. Regarding haemogregarines, these parasites have been found in the archipelago infecting lizards of the genera *Gallotia* [38–43] and *Tarentola* [44]. However, in most cases detection was based on microscopy, and only one work suggests different haemogregarine morphological forms infecting *Gallotia* [37]. In the current study, we use microscopy and phylogenetic approaches to analyze the diversity, distribution and host specificity of haemogregarines infecting the Canarian lizard communities. We sampled the three native lizard groups (*Tarentola*, *Chalcides* and *Gallotia*) across the archipelago. These different lizard genera present multiple colonization events of the Canary Islands [45–47] (Fig. 1) and can occur on the same localities in close proximity. In fact, there are reports of *Gallotia* spp. consuming members of the other lizard genera, along with conspecifics and other vertebrates [48–51]. Given these factors, the regarded low host specificity of haemogregarines, their alternative modes of transmission, and what has been described for insular parasites [19, 20], all conditions seem to favor the occurrence of host-switches and the use of multiple hosts (i.e. low specificity). So, past-punctual or still-present expansions of the host range of Canarian haemogregarines are expected. Additionally, as the Canary Islands are a biodiversity hotspot with high levels of endemism [52], we also expect to find new haemogregarine haplotypes unique to the archipelago.

## Methods

### Study area and hosts

The Canary Islands are a volcanic archipelago located 100 km off the northwest African coast (Fig. 1a). They have a well-known geological genesis, with a chronological east-west island emergence ranging from 20.6 to 1.12 Ma [52, 53]. Despite their proximity and considerable age, these islands were never connected to the mainland, and native fauna and flora originated from long-distance dispersals from Africa, Europe or other archipelagos [54]. The current reptile endemic fauna includes 16 native lizard species of three genera, each belonging to a different family. The three lizard groups can be found inhabiting the same locations, though they differ in their ecology: *Gallotia* lacertids are diurnal and ground-dwelling; *Chalcides* skinks are diurnal but semi-fossorial; and *Tarentola* geckos are crepuscular/nocturnal and saxicolous [55]. *Gallotia* ancestors colonized the archipelago shortly after the first island emerged [45]. This happened in a single event from the mainland (about 17 to 20 Ma), followed by gradual expansion and diversification through all the seven main islands (Fig. 1b). While eastern and central islands are inhabited by a single species each, the western islands harbor representatives of two other *Gallotia* lineages: one of giant lizards (all critically endangered or extinct), and another of smaller, common species. By contrast, the two other lizard groups arrived in multiple colonization events. The *Chalcides* skinks reached the archipelago from the mainland twice [46]: first to the western islands and Gran Canaria (around 7 Ma); and later to the eastern group (Fuerteventura and Lanzarote, 5 Ma) (Fig. 1c); and never colonized La Palma. The *Tarentola* geckos present the most complex history, with three independent colonizations [47] (Fig. 1d). The first established in the eastern islands (about 5.3–11.2 Ma), the second in Tenerife, La Gomera and La Palma (dated 4.1 to 8 Ma), and the third in Gran Canaria and El Hierro (5.3–6.7 Ma). It should however be noted that successive waves of colonization and ecological replacement may have taken place [56], with recent extinctions being known [57].

### Sample collection and parasite screening

**Fig. 2:**
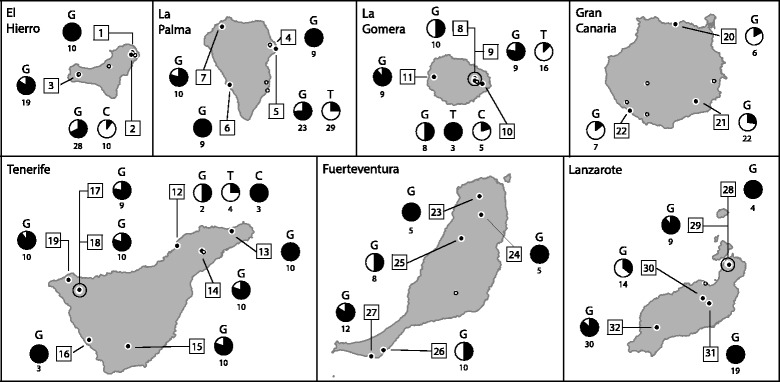
Prevalence variation across the sampling localities. For each locality, pie charts illustrate the proportion of infected (black) and uninfected (white) individuals for each genus and underneath are the corresponding number of sampled individuals. The numbers within squares represent the sampling locality codes (the same as used in the text and Additional file 1: Table S1, where further information is available). *Abbreviations*: G, *Gallotia*; T, *Tarentola*; C, *Chalcides*)

Eleven of the sixteen Canarian lizard species were sampled for this study, as it was not possible to assess the endangered ones (*Gallotia bravoana*, *Gallotia simonyi*, *Gallotia intermedia*, *Gallotia auaritae* and *Chalcides simonyi*). Sampling was carried out under permits by the corresponding island authorities. Blood slides from 825 individuals of the three host genera (406 *Gallotia*, 266 *Tarentola* and 153 *Chalcides*) were collected from 46 locations across the seven main Canary Islands in several field trips between 2009 and 2014 (Fig. 2, Additional file 1: Table S1). In 27 of these locations, lizards of different genera occurred in sympatry (14 locations with *Gallotia* and *Tarentola*, two with *Gallotia* and *Chalcides*, two with *Tarentola* and *Chalcides*, and nine with the three genera). Blood was collected on slides for microscopy observation and on Whatman paper for genetic characterization, along with tail-tip host tissue samples which were used when the blood on paper was not available (for more details, see [58]). Additionally, 54 samples from the genus *Psammodromu*s from North Africa and the Iberian Peninsula, the closest known relative of the genus *Galloti*a [59], were also screened.

Blood slides were examined at 400× magnification, under an Olympus CX41 microscope with an in-built digital camera (SC30) (Olympus, Hamburg, Germany). All slides were initially screened for the presence of haemogregarines to assess their prevalence (i.e. the percentage of samples infected with haemogregarines). Each slide was examined until an infected cell was detected or for a minimum of 5 min (approximately ranging between 5–15 min) [41], after which the sample was considered as negative. Parasitaemia levels were then estimated for the infected samples, and calculated as the percentage of haemogregarine-infected cells per 2500 erythrocytes. For such, five random areas of each slide were photographed at 400× magnification with the cell^B software (Olympus, Münster, Germany), and 500 erythrocytes per area were counted using the Cell Counter plugin from the image processing software ImageJ v. 1.50b [60]. In 5.3% of the positive cases parasitaemia could not be estimated due to poor slide quality. Differences in overall prevalence between host genera and islands were estimated using a Generalized Linear Model (GLM) with a binomial distribution, with prevalence as the dependent variable and host genera and island as explanatory variables. Given that not all host lizard genera are present in all the islands, the interaction effect between the two explanatory variables was not considered. A second GLM analysis was performed for the *Gallotia* subset to confirm the differences in prevalence among islands. Analyses were performed in R version 3.3.1 [61].

### Molecular identification and genetic analyses

Molecular characterization was performed on a subset of the infected samples. For *Gallotia*, up to five random representatives from each infected population were extracted (plus some extra individuals were sequenced to confirm parasite identification). Moreover, all infected *Tarentola* and *Chalcides* samples were also analyzed (although for one *Chalcides* individual, all PCR amplification attempts were unsuccessful). DNA from host blood or tissue was extracted using standard high-salt methods [62]. The PCR reactions were performed using primers specific for a 600 bp long region of the *18S* rRNA gene, HepF300 and HepR900 [63]. For details on PCR conditions, see Harris et al. [64]. The *18S* rRNA gene remains the most used genetic marker for the majority of haemogregarine clades [27, 65]. The pair of primers was chosen given its higher amplification success across different haemogregarine lineages infecting reptiles when compared to other primers (e.g. HEMO1 and HEMO2 [66], and EF and ER [67]), and because it provides comparable results to longer sequences in phylogenetic analysis [68]. Efforts to amplify other genetic markers available for haemogregarines [65], namely other fragments of the *18S* rRNA gene [66, 67] and the ITS1 region [69], were unsuccessful. The amplified products were purified and sequenced by an external company (Beckman Coulter Genomics, UK).

Sequences were compared to the GenBank database to confirm the identity of the amplified products using the NCBI nucleotide BLAST. A total of 137 haemogregarine sequences were obtained from *Gallotia* hosts, 12 from *Tarentola* and four from *Chalcides*. Sequences were corrected and aligned in Geneious v5.6.7 [70], using the MAFFT algorithm [71]. Clean high-quality sequences were first used to identify the distinct haplotypes present, then the sequences of lower quality and with ambiguities were compared to these to ascertain their identity. Cases of sequences with double peaks were consistent with the previously identified haplotypes and are regarded as mixed infections. Uncorrected pairwise distances (p-distances) between haplotypes were calculated in MEGA7 [72], using a 569 bp alignment of the nine new haplotypes (Additional file 2: Table S2). New haplotype sequences are deposited in the GenBank database, under the accession numbers MG787243-MG787253.

For the phylogenetic analyses, 137 GenBank sequences of other haemogregarines were added (Additional file 3: Table S3). As putative outgroups of the Canarian haemogregarines, sequences of specimens infecting *Chalcides*, *Tarentola* and other lizard species from the African and Iberian mainland were included (previously assessed in [44, 68, 73, 74]), as well as the two new sequences from the host genus *Psammodromus*, the closest relative to *Gallotia*. In accordance with Barta et al. [29], *Haemogregarina balli* and *Dactylosoma ranarum* were used as outgroups for the overall phylogeny. In total, the final alignment matrix included 148 sequences and 583 nucleotide positions. The substitution model of evolution was chosen according to the BIC criterion selected by jModelTest 2 (model TIM1+I+G) [75]. Phylogenetic relationships were estimated using maximum likelihood (ML) and Bayesian inference (BI) methods. ML analysis was performed in PhyML 3.1 [76], with nodal support estimated using the bootstrap technique [77] with 1,000 replicates. For the BI analysis, MrBayes v.3.2.6 [78] was used, with parameters estimated as part of the analysis. The analysis was run for 1 × 10^7^ generations, saving 1 tree each 1000 generations. The log-likelihood values of the sample point were plotted against the generation time and all the trees prior to reaching stationarity were discarded as ‘burn-in’ samples (25%). Remaining trees were combined in a 50% majority consensus tree, in which frequency of any particular clade represents the posterior probability. Additionally, phylogenetic networks for the clades containing Canarian haemogregarines were constructed using the statistical parsimony approach implemented in TCS [79] and displayed graphically using tcsBU [80].

A hierarchical analysis of molecular variance (AMOVA) was performed to test the hypotheses regarding genetic structure among haemogregarine haplotypes, using islands and host species as variables, and locations within these. Analysis was run both in the software Arlequin version 3.5.2 [81] and in the *poppr* package [82] in R [61], which implements the AMOVA tests from the packages *ade4* [83] and *pegas* [84], with 1000 Monte Carlo permutations in all cases to assess statistical significance.

## Results

### General assessment of haemogregarine prevalence and parasitaemia

n total, 301 specimens (36.4% of the individuals sampled) from 32 locations (69.6% of the localities) were infected with haemogregarines (see Fig. 2, Additional file 1: Table S1, Additional file 4: Table S4). The three reptile groups differed in total prevalence values (*χ*^2^ = 399.28, *df* = 2, *P* < 0.001), with the genus *Gallotia* being the most commonly infected (69.7%). By contrast, only 4.9% of *Tarentola* and 3.3% of *Chalcides* individuals were parasitized with haemogregarines. We also found a significant effect of the factor island (*χ*^2^ = 120.44, *df* = 6, *P* < 0.001). Differences of prevalence between islands were also observed when testing only the dataset of *Gallotia* spp. (*χ*^2^ = 31.44, *df* = 6, *P* < 0.001), with Gran Canaria showing the lowest infection levels (18.2%) while Tenerife had the highest (84.4%, Additional file 4: Table S4). As for parasitaemia (see Additional file 4: Table S4), *Gallotia* and *Tarentola* had similar mean values (1.07%), although the range for the former was wider (0.02–29.48 *vs* 0.02–4.36%, respectively). In *Chalcides*, parasitaemia was lower (mean = 0.05%, range = 0.02–0.16%).

### Haemogregarine diversity and phylogenetic relationships

**Fig. 3:**
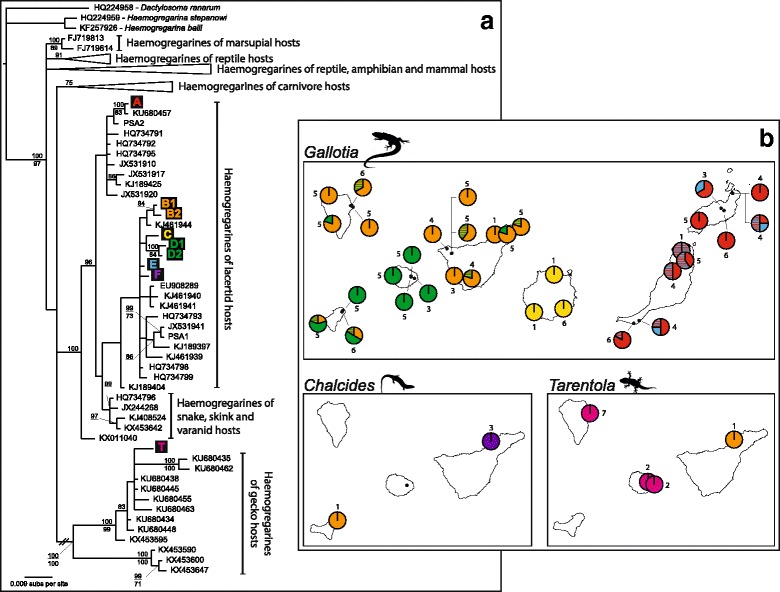
Phylogenetic analysis (**a**) and distribution (**b**) of the haemogregarine haplotypes infecting the three lizards studied. Evolutionary relationships were estimated using the *18S* rRNA gene. Presented is the tree derived from the BI analysis. Bayesian posterior probabilities are given above nodes and bootstrap values for ML below them (only values above 75 for the former and 70 for the latter are shown). For better reading, some branches of the phylogenetic tree have been collapsed or shortened (Additional file 3: Table S3 contains all the GenBank codes and extra information on the sequences used). Sequence KU680457, identified with an asterisk in the tree, corresponds to the infection in *T. angustimentalis* previously reported by Tomé et al. [46]. The nine Canarian haplotypes are differentiated by colors in the tree and maps (here the striped pattern identifies mixed infections). Pie charts represent the proportion of each haplotype per location, and beside them is the number of sequenced infections

In total, nine haplotypes (A, B1, B2, C, D1, D2, E, F and T) were found infecting the Canarian lizards. The phylogenetic analysis showed congruent patterns for the ML and BI analyses. Haplotypes grouped into two well-supported clades (Fig. 3a). The first includes only Haplotype T, which clusters within a recently discovered clade of haemogregarines infecting geckos from Morocco, Algeria and Oman. Haplotype T was only found in three localities from La Palma and La Gomera, with an overall prevalence of 19.4%. Haplotype T diverged by 0.058 (uncorrected p-distance) from the other Canarian haplotypes. The other eight haplotypes (haplotypes A to F) are part of a second ‘*Karyolysus*’ clade (*sensu* Karadjian et al. [27]) and are further distributed in two subclades (Fig. 3a). Haplotype A is placed in a subclade together with haemogregarines infecting lizards from the Iberian Peninsula and North-western Africa, while the second subclade comprises the remaining haplotypes (p-distance = 0.0018 between haplotype A and haplotypes B1 to F). This second subclade comprises haemogregarines from a wide range of reptile hosts, including lacertid lizards, snakes and skinks from Europe, Morocco and Oman. Unfortunately, the relationships within this subclade are not highly supported (Fig. 3a). The highest p-distance within this group was 0.011 between B2 and D1, and the lowest was 0.002 (1 mutation) between B1 and B2, and also D1 and D2 (see Additional file 2: Table S2). Given the sequence similarity and association between them, we treat these sister haplotypes as a single unit each and hereafter they will be called haplogroups B and D when referred to collectively. Regarding the parasites from *Gallotia*’s sister taxa, two haemogregarine haplotypes were discovered in *Psammodromus algirus* from Morocco (identified as PSA 1 and 2 in Fig. 3a and Additional file 5: Figure S1a). One grouped within the same subclade as haplotype A, while the other one clustered with the remaining haplotypes found in *Gallotia* spp., although with low support. Haplotype networks showing the relationships among haplotypes are detailed in Additional file 5: Figure S1.

### Geographical distribution of haplotypes and host specificity

All recovered haplotypes were restricted to a specific lizard host genus. Seven of them (haplotypes A, B1, B2, C, D1, D2 and E) infected *Gallotia* lizards, while haplotype F was exclusive to *Chalcides* and haplotype T to *Tarentola* (Fig. 3b, Additional file 1: Table S1). The exception was haplotype B1 which, although mostly found in *Gallotia*, was also detected in two instances infecting *Chalcides* (one individual, locality 2) and *Tarentola* (one individual, locality 12). Haplotype F was found in a single site in Tenerife (location 2), infecting three *Chalcides* individuals. Haplotype T was found in *Tarentola* from two islands (La Palma and La Gomera). In *Gallotia*, haplotypes A and E were found in the two easternmost islands, haplotype C in the central island of Gran Canaria, and haplotypes B1, B2, D1 and D2 in the western islands. AMOVA tests were performed on the set of haplotypes with a plausible common origin: B1, B2, C, D1, D2 and E. Results were the same for the three different methodologies and showed a high correlation of genetic distances to both islands (69.32% of molecular variance) and *Gallotia* host species (72.82%) (Table 1).

**Table 1:** AMOVA tests of genetic structure for haplotypes B1 to E

Source of variation	*df*	SS	Var. comp.	Var. %	ɸ-statistic^a^	*P-*value^b^
By island						
Among islands	6	107.70	1.12	69.32	ɸ_ST_ = 0.65	>> 0.001
Among locations within islands	22	6.86	-0.06	-3.90	ɸ_SC_ = -0.13	0.68
Within locations	89	49.74	0.56	34.58	ɸ_CT_ = 0.69	>> 0.001
By *Gallotia* spp.						
Among host species	3	104.19	1.40	72.82	ɸ_ST_ = 0.71	>> 0.001
Among locations within host spp.	25	10.38	-0.04	-1.87	ɸ_SC_ = -0.69	0.52
Within locations	89	49.74	0.56	29.05	ɸ_CT_ = 0.73	>> 0.001

^a^The ɸ-statistics indicate the degree of genetic differentiation within and among groups in relation to the total (i.e., zero would be a situation of panmixia and one total separation)

^b^*P*-values were calculated from 1000 permutations

*Abbreviations*: df, degrees of freedom; SS, sums of squares; Var. comp., variance components; Var. %, percentage of variation

Results from the analysis performed in the Arlequin software, by island and by *Gallotia* host species. Numbers have been reduced to two decimals for presentation

It should also be noted that *Gallotia* individuals presented mixed infections (in 13 of the 32 localities with infected *Gallotia*, see Additional file 1: Table S1 and Fig. 3b). From the island perspective, the number of haplotypes present varied (Fig. 3b). Gran Canaria and La Gomera had only one haplotype each (C and D1, respectively). Two haplotypes were present in Fuerteventura, Lanzarote (A and E) and El Hierro (B and D1). Four haplotypes were found in Tenerife: B1, B2, D1, together with F infecting *Chalcides*. Finally, La Palma harbored the highest number of haplotypes, with haplotypes B1, B2, D1 and D2 infecting *Gallotia* and T infecting *Tarentola*.

### Differences in prevalence and parasitaemia

Because only a subset of parasite specimens was sequenced for *Gallotia*, we could not thoroughly assess the prevalence for the different haplotypes. However, as sequenced samples were randomly selected, the relative frequency between sympatric *Gallotia* haplotypes gives a reasonable approximation of their prevalence (Additional file 1: Table S1 and Fig. 3b). Within the eastern islands (Lanzarote and Fuerteventura) haplotype A was more frequent than haplotype E. In the Central island of Gran Canaria, only haplotype C was present, in a very low frequency (18.2%). In both Tenerife and La Palma, haplotype B was more frequent than haplotype D, while in El Hierro the reverse occurred. Lastly, haplotype D was the only one present in La Gomera, in a lower frequency (52.2%) than the overall mean prevalence in the genus *Gallotia* (69.7%). Regarding parasitaemia, haplotype A had the highest mean (1.15%) and E the lowest (0.13%), though it should be noted that sample size was quite disparate between haplotypes (Additional file 4: Table S4). Haplotype B was the only one for which cross-infections were detected, having a mean parasitaemia of 1.38% in the genus *Gallotia*, while for the only two cases of *Chalcides* and *Tarentola* infected with this haplotype, values were 0.02% and 0.04%, respectively.

## Discussion

### Haemogregarine distribution and transmission

This is the first comprehensive overview on the haemogregarines infecting the endemic lizards of the Canary Islands. This archipelago is considered a biodiversity hotspot due to its high levels of endemism [52] and our results further support this, as we identified a high diversity of haemogregarine haplotypes previously unknown, none matching sequences from other geographical areas. Moreover, and although sampling included host sympatric localities, haplotypes were mostly restricted to a particular lizard genus, suggesting that these parasites are highly specific to their vertebrate hosts. Seven of the nine haplotypes were found infecting a single host species (haplotype C in *Gallotia stehlini*, E in *Gallotia atlantica*, and F in *Chalcides viridanus*) or species with a recent common ancestry (haplotype D in *Gallotia galloti* and *Gallotia caesaris*, and haplotype T in *Tarentola gomerensis* and *Tarentola delalandii*). In contrast, haplotype B1, which mostly infected western *Gallotia*, was also found in *Tarentola* and *Chalcides*. Similarly, haplotype A, in this study frequently found in *G. atlantica* (but not geckos), was previously reported infecting one *Tarentola angustimentalis* from Fuerteventura [44]. Given the low prevalence of these two haplotypes in *Tarentola* and *Chalcides* hosts, we consider these cases as occasional cross-infections (although these lizards might still be competent hosts, as peripheral blood stages were observed). This also highlights that, to correctly assess host range and specificity of a parasite, studies should extend to the community of potential sympatric hosts [9, 15, 85]. Regarding mixed infections, interestingly the few cases confirmed were only detected in *Gallotia* hosts (Fig. 3 and Additional file 1: Table S1). These cases involved only *Gallotia* specific haplotypes that co-occurred in the same island, although some of them were distantly related (A and E).

It has been suggested that haemogregarines are more host-specific to their invertebrate hosts than to their vertebrate hosts [22, 29]. This is relevant as the level of specialization of the vector is expected to condition the opportunity of a parasite to encounter a given vertebrate host [86, 87]. Mites of the genus *Ophionyssus* are the main suspected vector and definitive host of the “*Karyolysus*” group ([21, 88, 89], and references within), although this has not been confirmed as general. In the Canary Islands, three *Ophionyssus* species have been described parasitizing *Gallotia* spp. [90, 91], and their role as vector for haemogregarines has already been suggested [36]. Their distribution seems congruent with the observed patterns for haemogregarine haplotypes from *Gallotia*, with a species reported for eastern, Gran Canaria, and western islands. Additionally, Bannert et al. [92] examined the three lizard groups in Tenerife, and found neither geckos nor skinks infected by *Ophionyssus* spp. (while these were highly prevalent in *Gallotia* lizards of the same location). On the other hand, *Tarentola* from the Canary Islands are reported to be infected by mites of the genus *Geckobia* [93], a specialist mite of geckos [94], although again, their role as vectors of haemogregarines has not been confirmed. Given the high dependency of mites on the lizard host for their life-cycle [92, 94], it seems reasonable to assume that they colonized the archipelago while carried by their lizard hosts, possibly along with the haemogregarines. Surely, the colonization and expansion success of their invertebrate hosts must have also conditioned the distribution of the haemogregarines. Therefore, future work should identify and assess the role of these vectors in the colonization success and adaptation to new environments, as well as in the host specificity of parasites with heteroxenous life-cycles. On the other hand, *Gallotia* spp. are known to consume other vertebrates, including conspecifics [48–51], which might provide an additional infection pathway [95]. Indeed, transmission of haemogregarines *via* predation has been shown in other hosts [24, 25]. Also worth noting, in *Gallotia* lizards, *Sarcocytis gallotiae* has adapted to insularity by using the same host species as both definitive and intermediate host by exploiting its cannibalistic behavior [96, 97].

### Phylogenetic relationships and biogeography

Canarian lizards share common ancestors with lizards from North Africa and the Iberian Peninsula [45–47], so it is likely that their parasites share this origin and our results support this. In fact, the haplotypes detected here clustered in haemogregarine clades found in European and African reptiles (Fig. 3a). Particularly, some of them were related to haemogregarines from the hosts’ closest relatives, such as the genus *Psammodromus* and other *Tarentola* species from the continent. The pattern is, however, not so clear for the haemogregarines infecting skinks (haplotype F). Nonetheless, further conclusions are difficult due to the low support of the phylogenetic relationships within this group. Additionally, our results provide preliminary insights on how these haemogregarines colonized the Canary Archipelago. We note, however, that some inferences remain preliminary as additional data are necessary, namely the identity of haemogregarine definitive hosts, the haplotypes infecting the endangered Canarian lizard species, and phylogenetic information from additional faster-evolving molecular markers.

Our results suggest that haemogregarines arrived to the Canary Islands along with the ancestors of the three lizard genera. The haemogregarine with the clearest colonization is haplotype T. The ancestors of one lineage of *Tarentola* arrived from the African mainland to the Canary Islands, reaching first Tenerife and then dispersing to La Palma and La Gomera (Fig. 1d) [47]. The haemogregarine ancestors of haplotype T might have followed the same path, and its absence in Tenerife could be explained by extinction events or low detectability as a consequence of low prevalence values. Regarding haemogregarines infecting *Gallotia*, haplotypes A and E were found in the two easternmost islands. These were the first islands of the archipelago colonized by *Gallotia*’s ancestors (Fig. 1b) [45]. Although haplotypes A and E belong to two distinct clades, they could have “shared the boat”, colonizing these islands in the same event aboard their *Gallotia* ancestor hosts (and/or vector hosts). The absence of haplotype A (or any close relatives) on the remaining islands suggests that it “missed the boat” when *Gallotia* further expanded through the archipelago (or went extinct during these new colonizations). Contrarily, haplotype E has closely related haplotypes (B, C and D) in the other islands, supporting further colonizations of this haplotype ancestor to other islands together with its *Gallotia* host. Unfortunately, phylogenetic relationships within this clade are not fully resolved, preventing us from drawing more conclusions. The results from the AMOVA show a high level of genetic structure concordant with both *Gallotia* host species and geography, supporting the hypothesis that these haplotypes followed the colonization paths of their *Gallotia* hosts. Finally, regarding haplotype F, which was detected in skinks from Tenerife, the simplest scenario would be that it arrived there along with the *Chalcides* ancestors that colonized the western islands (Fig. 1c) [46], and then either did not further expand or later retracted (as it was only found in one location). Alternatively, host-switching events might have taken place, from the skinks to the *Gallotia* or vice-versa, although with the current data it is not possible to infer if this is the case. The presence of paralogs is a characteristic of the *18S* rRNA gene that might cloud the interpretation of phylogenetic results, and it has been confirmed for some apicomplexans (e.g. [98–100], suspected for haemogregarines in [101]). However, the haemogregarine haplotypes detected here present a non-random distribution (as shown by the aforementioned geographical structuration) and, although we did find a few cases with more than one haplotypic form within the same host individual, these were instead more suggestive of mixed infections (as they were rare and correspond to the haplotypes retrieved in single infections). Given these reasons, we do not suspect paralogy is an issue in the current dataset, though this can only be ruled out completely when other sequence data are available for these parasites, for example from mitochondrial genes.

### Factors shaping haemogregarine diversity and host specificity

Parasite prevalence, parasitaemia and genetic diversity were higher in *Gallotia* hosts than in *Tarentola* and *Chalcides* (values in *Gallotia* spp. were consistent with previous studies [39–43]). Interestingly, a similar trend was also observed in nematodes of the genus *Spauligodon* infecting the same hosts [37]. Such congruence for two radically different parasites might suggest that host ecological characteristics play a role in parasite abundance, diversity distribution and transmission dynamics [31, 102], alongside parasite adaptation or host immunology. The three lizard genera can be found inhabiting the same localities but differ in daily activity and microhabitat use [55]. This segregation and differences in host densities might lead to heterogeneity in the encounter opportunity, affecting the chances of parasite transmission and expansion [103–105]. For example, Martín et al. [106] have conjectured that a fossorial lifestyle (such as in *Chalcides* [46]) could lead to a lower exposure to vectors, and consequently to haemogregarines. The lizard groups also differ in their body temperatures, with *Gallotia* lizards thermoregulating at higher temperatures than *Tarentola* geckos [107, 108]. Host internal temperature might be also relevant since the infection levels of haemogregarines seem to be affected by this factor [109]. Additionally, distinct lizard populations are exposed to different environmental stressors (either natural climatic or human related), which might locally influence prevalence and parasitaemia, e.g. [110]. Nonetheless, nematodes and haemogregarines did not show the same patterns of diversity distribution and potential colonization pathways. While both *Spauligodon* nematodes and haemogregarines show high levels of diversity and host specificity, past host-switch events were detected for the former, but not for haemogregarines. The two parasites differ in many characteristics; most notably *Spauligodon* nematodes have a direct life-cycle while haemogregarines have a multiple-host one. This might have relevant repercussions, as a heteroxenous life-cycle complicates the maintenance of the transmission dynamics, and increases the constraints for a successful establishment ([95, 111] but see [15]).

There is a marked contrast in the number of haemogregarine haplotypes between eastern (Gran Canaria, Fuerteventura and Lanzarote) and western islands (El Hierro, La Palma, La Gomera and Tenerife). This is partly due to the presence of the haplotypes infecting skinks and geckos but, considering only the haplotypes infecting *Gallotia* lizards, this pattern is still maintained. There is a lower *Galloti*a species richness in the East (only two species), compared to the radiation that took place in the West (in total seven species of two distinct lineages [45]). Here, co-speciation with the host [112] and/or isolation in the different western islands might have fomented parasite diversification (as also hypothesized for nematodes by Jorge et al. [113]). On the other hand, an impoverished biodiversity in the eastern islands relative to the rest of the archipelago is a general pattern [114, 115], which has also been observed in the helminth fauna infecting *G. atlantica* [116], and relates to the nonlinear relation between the age of an oceanic island and its biodiversity [117, 118]. Also, Fuerteventura and Lanzarote were connected several times during the Pleistocene [119, 120], which would dilute the effect of their previous isolation. Within-island geological events could explain the heterogeneity in prevalence and in the relative abundance of the parasite haplotypes. For example, volcanic eruptions and landslides have led to repetitive isolations and secondary contacts of host populations [121–123]. Another possible phenomenon is parasite spillover from now-extinct or introduced hosts [124], as insular systems are particularly prone to extinction-recolonization events and to introductions [17, 125]. However, we found no evidence of this in the current results, given the congruence between haemogregarine and host distributions, and the fact that we did not identify parasite haplotype exchange between islands with known lizard translocations or introductions [126].

Our results showed that haemogregarines infecting the lizards of the Canary Island did not have a low host specificity or represent switching events between host groups. This contrasts with our initial predictions, as it does not seem to support the hypothesized parasite niche broadening expected in insular systems [19]. Although such might have still occurred, it is not evident based on the currently available data. While the mechanism behind the high host specificity pattern of these haemogregarines is not elucidated, we list some of the factors that might be involved. Segregation between lizard groups due to their ecology and differences in transmission (such as their vectors) seem major suspects, but molecular factors may (also or instead) play a role, including host immunity response or the ability of the parasite to exploit host resources [127]. It should however be noted that occasional infections show that the different lizard groups might be competent hosts, at least for some of the Canarian haemogregarine haplotypes. Thus, even if these haemogregarines can have a lower specificity regarding their potential host range, their realized niche seems to actually be restrictive in the hosts used. This is relevant as haemogregarines are generally considered as having low host specificity for their vertebrate hosts [22, 29, 128]. This has been supported by experimental studies (e.g. [69], references within [129]), but might not reflect the reality in the wild [129]. Additionally, it should be noted that host specificity and host-switch potential might depend on parasite lineage [31, 37], so the patterns might differ between haemogregarine groups and explain incongruences across studies.

## Conclusions

This is the first comprehensive study on haemogregarines of the Canary Islands, specifically those infecting lizards. Our results seem to suggest a high level of host specificity for haemogregarine parasites infecting lizards of the Canary Islands, with no evidence of host-switching events. Moreover, for haemogregarines infecting *Gallotia* hosts, co-diversification apparently took place, as a high diversity of geographically-structured parasite haplotypes is present. The possible factors shaping the host specificity and biogeography of these parasites include differences in host ecology, vector identity and parasite transmission. The observed patterns contrast with our initial expectations, and similarly to *Spauligodon* nematodes, no long-lasting host range broadening was observed. This highlights how host specificity depends on parasite characteristics and evolutionary history, and questions what is generally assumed for insular parasites and haemogregarines. Lastly, we note that the nine parasite haplotypes found in the Canary Islands are distinct from those detected until this date on the mainland. Although more markers and samples are needed to fully confirm our results, the evolutionary history inferred with the *18S* rRNA gene suggests that the Canarian haemogregarines underwent a strong diversification process, representing one more example of the high diversity present in this archipelago.

## Additional files

Additional file 1: **Table S1.** Sampling locations and prevalence values. (DOCX 31 kb)

Additional file 2: **Table S2.** Uncorrected p-distances between the nine haemogregarine haplotypes discovered in the Canarian lizards. (DOCX 13 kb)

Additional file 3: **Table S3.** GenBank codes and additional information on the genetic sequences used for the phylogenetic analyses. (DOCX 26 kb)

Additional file 4: **Table S4** Statistical summary of the variation of haemogregarine prevalence and parasitaemia from Canarian lizards. (DOCX 18 kb)

Additional file 5: **Figure S1.** Networks of the haemogregarine haplotypes infecting *Gallotia*, *Chalcides* (a) and *Tarentola* lizards (b). Represented is the network estimations performed with TCS. Black nodes represent mutations, while coloured ones correspond to the sequences of Canarian haemogregarines (colours match the ones used in Fig. 3), and the white ones correspond to the remaining samples. The network of a includes the samples of the haemogregarine clade from lacertid, snake, skink and varanid hosts (as identified in Additional file 4: Table S4) and b includes the samples of the clade from gecko hosts. (PDF 911 kb)

## Abbreviations

AMOVA: Analysis of molecular variance
BI: Bayesian inference
BIC: Bayesian information criterion
DNA: Deoxyribonucleic acid
GLM: Generalized linear model
ITS1: Internal transcribed spacer 1
ML: Maximum likelihood
PCR: Polymerase chain reaction
rRNA: Ribosomal ribonucleic acid
