# Supplementary material for: Along for the ride or missing it altogether: exploring the host specificity and diversity of haemogregarines in the Canary Islands
Source: Parasit Vectors. 2018 Mar 19;11:190. doi: 10.1186/s13071-018-2760-5 (PMC5859493; doi:10.1186/s13071-018-2760-5)
Supplement: Supplementary file 1 — Table S1. Sampling locations and prevalence values. (DOCX 31 kb) [file 13071_2018_2760_MOESM1_ESM.docx]

**Additional file 1: Table S1.** Sampling locations and prevalence values.

|  | |  |  |  | ***Gallotia*** | | | ***Tarentola*** | | | ***Chalcides*** | | |
| --- | --- | --- | --- | --- | --- | --- | --- | --- | --- | --- | --- | --- | --- |
| **Island** | Location | Lat. | Lon. | Loc. | Screened | Positives | Sequenced | Screened | Positives | Sequenced | Screened | Positives | Sequenced |
| **El Hierro** | |  |  |  | **57** | **45** | **16 (3 B1; 2 B1/D1; 11 D1)** | **17** | **0** | **0** | **36** | **1** | **1 (1 B1)** |
| La Lapa | | 27.76 | -18.00 |  | - | - | - | 3 | 0 | 0 | - | - | - |
| North Valverde | | 27.82 | -17.91 | 1 | 10 | 10 | 5 (5 D1) | - | - | - | - | - | - |
| Villa de Valverde | | 27.81 | -17.91 | 2 | 28 | 19 | 6 (2 B1; 1 B1/D1; 3 D1) | - | - | - | 10 | 1 | 1 (1 B1) |
| East Valverde | | 27.81 | -17.90 |  | - | - | - | 10 | 0 | 0 | 10 | 0 | - |
| N. Sra. de los Reyes | | 27.73 | -18.12 | 3 | 19 | 16 | 5 (1 B1; 1 B1/D1; 3 D1) | 4 | 0 | 0 | 9 | 0 | - |
| Camino de la Virgen | | 27.73 | -18.12 |  | - | - | - | - | - | - | 7 | 0 | - |
| **La Palma** | |  |  |  | **53** | **43** | **21 (16 B1; 1 B2; 1 B1/B2; 2 B1/D1; 1 D2)** | **33** | **7** | **7 (7 T)** | **0** | **0** | **0** |
| El Brasil | | 28.75 | -17.75 |  | - | - | - | 1 | 0 | 0 | - | - | - |
| La Lomada | | 28.75 | -17.75 | 4 | 9 | 9 | 6 (3 B1; 1 B2; 2 B1/D1) | 2 | 0 | 0 | - | - | - |
| Piedra Alta | | 28.73 | -17.73 | 5 | 23 | 17 | 5 (4 B1; 1 B1/B2) | 29 | 7 | 7 (7 T) | - | - | - |
| Callejones | | 28.60 | -17.77 |  | - | - | - | 1 | 0 | 0 | - | - | - |
| Playa Salemera | | 28.57 | -17.76 |  | 2 | 0 | 0 | - | - | - | - | - | - |
| Puerto Naos | | 28.59 | -17.91 | 6 | 9 | 9 | 5 (4 B1; 1 D2) | - | - | - | - | - | - |
| Santo Domingo | | 28.82 | -17.94 | 7 | 10 | 8 | 5 (5 B1) | - | - | - | - | - | - |
| **La Gomera** | |  |  |  | **46** | **24** | **18 (18 D1)** | **29** | **5** | **4 (4T)** | **15** | **1** | **-** |
| South Parque Majona | | 28.13 | -17.16 | 8 | 10 | 5 | 5 (5 D1) | - | - | - | - | - | - |
| El Jorado | | 28.11 | -17.15 |  | 10 | 0 | 0 | 10 | 0 | 0 | 10 | 0 | 0 |
| Langrero | | 28.10 | -17.13 | 9 | 9 | 7 | 5 (5 D1) | 16 | 2 | 2 (2 T) | - | - | - |
| El Atajo | | 28.12 | -17.16 | 10 | 8 | 4 | 3 (3 D1) | 3 | 3 | 2 (2 T) | 5 | 1 | 0 |
| Arure | | 28.13 | -17.32 | 11 | 9 | 8 | 5 (5 D1) | - | - | - | - | - | - |
| **Tenerife** | |  |  |  | **64** | **54** | **32 (24 B1; 2 B2; 1 B1/B2; 4 B1/D1; 1 D1)** | **43** | **1** | **1 (1 B1)** | **23** | **3** | **3 (3 F)** |
| Mesa del Mar | | 28.50 | -16.42 | 12 | 2 | 1 | 1 (1 B1) | 4 | 1 | 1 (1 B1) | 3 | 3 | 3 (3 F) |
| Risco Mogote | | 28.56 | -16.21 | 13 | 10 | 10 | 5 (4 B1; 1 B1/D1) | - | - | - | - | - | - |
| La Laguna | | 28.48 | -16.32 | 14 | 10 | 8 | 5 (3 B1; 1 B2; 1 D1) | - | - | - | - | - | - |
| S. Miguel de Geneto | | 28.48 | -16.32 |  | - | - | - | 10 | 0 | 0 | 10 | 0 | - |
| San Miguel | | 28.11 | -16.61 | 15 | 10 | 8 | 4 (3 B1; 1 B1/D1) | - | - | - | - | - | - |
| Las Cancelas | | 28.13 | -16.77 | 16 | 3 | 3 | 3 (3 B1) | 19 | 0 | 0 | - | - | - |
| Erjos de el Tanque | | 28.32 | -16.80 | 17 | 9 | 7 | 5 (2 B1; 1 B1/B2; 2 B1/D1) | - | - | - | - | - | - |
| Erjos | | 28.33 | -16.80 | 18 | 10 | 8 | 5 (5 B1) | - | - | - | - | - | - |
| Buenavista del Norte | | 28.37 | -16.85 | 19 | 10 | 9 | 4 (3 B1; 1 B2) | 10 | 0 | 0 | 10 | 0 | 0 |
| **Gran Canaria** | |  |  |  | **44** | **8** | **8 (8 C)** | **39** | **0** | **0** | **79** | **0** |  |
| San Andrés | | 28.14 | -15.56 | 20 | 6 | 1 | 1 (1 C) | - | - | - | 1 | 0 | 0 |
| Ingenio | | 27.92 | -15.41 |  | 1 | 0 | 0 | 10 | 0 | 0 | 10 | 0 | 0 |
| Aldea Blanca | | 27.84 | -15.48 | 21 | 22 | 6 | 6 (6 C) | 18 | 0 | 0 | 43 | 0 | 0 |
| Barranco de Arguineguín | | 27.79 | -15.67 |  | 4 | 0 | 0 | 10 | 0 | 0 | 10 | 0 | 0 |
| Playa Medio Almud | | 27.80 | -15.74 | 22 | 7 | 1 | 1 (1 C) | - | - | - | - | - | - |
| Barranco de Mógan | | 27.84 | -15.75 |  | 2 | 0 | 0 | - | - | - | - | - | - |
| Soria | | 27.91 | -15.67 |  | 2 | 0 | 0 | 1 | 0 | 0 | 15 | 0 | 0 |
| **Fuerteventura** | |  |  |  | **66** | **47** | **20 (11 A; 8 A/E; 1 E)** | **45** | **0** | **0** | **0** | **0** | **0** |
| Lajares | | 28.68 | -13.93 | 23 | 5 | 5 | 1 (1 A/E) | 2 | 0 | 0 | - | - | - |
| La Oliva | | 28.61 | -13.93 | 24 | 29 | 23 | 5 (2 A; 3 A/E) | 14 | 0 | 0 | - | - | - |
| Tefia | | 28.51 | -14.00 | 25 | 8 | 4 | 4 (2 A; 2 A/E) | 2 | 0 | 0* | - | - | - |
| Tuinege | | 28.30 | -14.02 |  | 2 | 0 | 0 | - | - | - | - | - | - |
| Esquinzo-Butihondo | | 28.08 | -14.31 | 26 | 10 | 5 | 4 (2 A; 1 A/E; 1 E) | 18 | 0 | 0 | - | - | - |
| Morro Jable | | 28.05 | -14.36 | 27 | 12 | 10 | 6 (5 A; 1 A/E) | 9 | 0 | 0 | - | - | - |
| **Lanzarote** | |  |  |  | **76** | **62** | **22 (18 A; 2 A/E; 2 E)** | **60** | **0** | **0** | **0** | **0** | **0** |
| Mirador del Rio | | 29.20 | -13.49 | 28 | 4 | 4 | 4 (4 A) | - | - | - | - | - | - |
| Yé | | 29.20 | -13.48 | 29 | 9 | 8 | 4 (1 A; 2 A/E; 1 E) | 2 | 0 | 0 | - | - | - |
| Caleta de Famara | | 29.12 | -13.58 |  | - | - | - | 10 | 0 | 0 | - | - | - |
| West Teguise | | 29.07 | -13.59 | 30 | 14 | 5 | 3 (2 A; 1 E) | 12 | 0 | 0 | - | - | - |
| Nazaret-Teguise | | 29.05 | -13.56 | 31 | 19 | 19 | 6 (6 A) | 11 | 0 | 0 | - | - | - |
| Yaiza | | 28.95 | -13.77 | 32 | 30 | 26 | 5 (5 A) | 25 | 0 | 0 | - | - | - |
| **Total** | |  |  |  | **406** | **283** | **137** | **266** | **13** | **12** | **153** | **5** | **4** |

Latitude and longitude coordinates are given for all locations, and where infections were found, a location number is attributed for identification in the text and Fig. 2. For the three lizard genera analyzed, the number of individuals sampled, infected and haemogregarines sequenced (between brackets is how many sequences corresponded to each parasite haplotype, including mixed infections) are given for each island and location. The asterisk in location 25 refers to the location where Tomé et al. [46] previously reported an infection in *T.* *angustimentalis*.
