# Supplementary material for: Along for the ride or missing it altogether: exploring the host specificity and diversity of haemogregarines in the Canary Islands
Source: Parasit Vectors. 2018 Mar 19;11:190. doi: 10.1186/s13071-018-2760-5 (PMC5859493; doi:10.1186/s13071-018-2760-5)
Supplement: Supplementary file 2 — Table S2. Uncorrected p-distances between the nine haemogregarine haplotypes discovered in the Canarian lizards. (DOCX 13 kb) [file 13071_2018_2760_MOESM2_ESM.docx]

**Additional file 2: Table S2.** Uncorrected p-distances between the nine haemogregarine haplotypes discovered in the Canarian lizards.

|  | **A** | **B1** | **B2** | **C** | **D1** | **D2** | **E** | **F** |
| --- | --- | --- | --- | --- | --- | --- | --- | --- |
| **B1** | 0.018 |  |  |  |  |  |  |  |
| **B2** | 0.019 | 0.002 |  |  |  |  |  |  |
| **C** | 0.018 | 0.005 | 0.007 |  |  |  |  |  |
| **D1** | 0.019 | 0.009 | 0.011 | 0.009 |  |  |  |  |
| **D2** | 0.018 | 0.007 | 0.009 | 0.007 | 0.002 |  |  |  |
| **E** | 0.016 | 0.005 | 0.007 | 0.005 | 0.007 | 0.005 |  |  |
| **F** | 0.018 | 0.004 | 0.005 | 0.007 | 0.009 | 0.007 | 0.005 |  |
| **T** | 0.058 | 0.058 | 0.056 | 0.053 | 0.060 | 0.058 | 0.058 | 0.060 |
