# Supplementary material for: Along for the ride or missing it altogether: exploring the host specificity and diversity of haemogregarines in the Canary Islands
Source: Parasit Vectors. 2018 Mar 19;11:190. doi: 10.1186/s13071-018-2760-5 (PMC5859493; doi:10.1186/s13071-018-2760-5)
Supplement: Supplementary file 3 — Table S3. GenBank codes and additional information on the genetic sequences used for the phylogenetic analyses. (DOCX 26 kb) [file 13071_2018_2760_MOESM3_ESM.docx]

**Additional file 3: Table S3.** GenBank codes and additional information on the genetic sequences used for the phylogenetic analyses.

| Phylogeny group | Accession code | Parasite | Host species | Host family | Host order | Host class | Country |
| --- | --- | --- | --- | --- | --- | --- | --- |
| **Outgroup** | HQ224958 | *Dactylosoma ranarum* | *Rana esculenta* | Ranidae | Anura | Amphibia | France |
|  | HQ224959 | *Haemogregarina balli* | *Chelydra serpentina* | Chelydridae | Testudines | Reptilia | Canada |
|  | KF257926 | *Haemogregarina stepanowi* | *Mauremys caspica* | Geoemydidae | Testudines | Reptilia | Iran |
| **Marsupials** | FJ719813 | *Hepatozoon* sp. | *Dromiciops gliroides* | Microbiotheriidae | Microbiotheria | Mammalia | Chile |
|  | FJ719814 | *Hepatozoon* sp. | *Dromiciops gliroides* | Microbiotheriidae | Microbiotheria | Mammalia | Chile |
| **Reptile hosts** | EU430231 | *Hepatozoon* sp. | *Amblyomma fimbriatum* | Ixodidae | Ixodida | Arachnida | Australia |
|  | EU430232 | *Hepatozoon* sp. | *Amblyomma fimbriatum* | Ixodidae | Ixodida | Arachnida | Australia |
|  | EU430236 | *Hepatozoon* sp. | *Hyalomma aegyptium* | Ixodidae | Ixodida | Arachnida | Algeria |
|  | JQ080303 | *Hepatozoon* sp. | *Aedes taeniorhynchus* | Culicidae | Diptera | Insecta | Ecuador |
|  | KC512766 | *Hemolivia* sp. | *Hyalomma aegyptium* | Ixodidae | Ixodida | Arachnida | Algeria |
|  | KF992698 | *Hemolivia mauritanica* | *Testudo graeca* | Testudinidae | Testudines | Reptilia | Turkey |
|  | KF992699 | *Hemolivia mauritanica* | *Testudo marginata* | Testudinidae | Testudines | Reptilia | Greece |
|  | KF992700 | *Hemolivia mauritanica* | *Testudo graeca* | Testudinidae | Testudines | Reptilia | Syria |
|  | KF992701 | *Hemolivia mauritanica* | *Testudo graeca* | Testudinidae | Testudines | Reptilia | Syria |
|  | KF992702 | *Hemolivia mauritanica* | *Testudo graeca* | Testudinidae | Testudines | Reptilia | Syria |
|  | KF992703 | *Hemolivia mauritanica* | *Testudo graeca* | Testudinidae | Testudines | Reptilia | Syria |
|  | KF992704 | *Hemolivia mauritanica* | *Testudo graeca* | Testudinidae | Testudines | Reptilia | Syria |
|  | KF992705 | *Hemolivia mauritanica* | *Testudo graeca* | Testudinidae | Testudines | Reptilia | Syria |
|  | KF992706 | *Hemolivia mauritanica* | *Testudo graeca* | Testudinidae | Testudines | Reptilia | Syria |
|  | KF992707 | *Hemolivia mauritanica* | *Testudo graeca* | Testudinidae | Testudines | Reptilia | Syria |
|  | KF992708 | *Hemolivia mauritanica* | *Testudo graeca* | Testudinidae | Testudines | Reptilia | Syria |
|  | KF992709 | *Hemolivia mauritanica* | *Testudo graeca* | Testudinidae | Testudines | Reptilia | Syria |
|  | KF992710 | *Hemolivia mauritanica* | *Testudo marginata* | Testudinidae | Testudines | Reptilia | Greece |
|  | KF992711 | *Hemolivia mariae* | *Egernia stokesii* | Scincidae | Squamata | Reptilia | Australia |
|  | KF992712 | *Hemolivia mariae* | *Egernia stokesii* | Scincidae | Squamata | Reptilia | Australia |
|  | KF992713 | *Hemolivia* sp. | *Rhinoclemmys pulcherrima* | Geoemydidae | Testudines | Reptilia | Nicaragua |
|  | KF992714 | *Hemolivia* sp. | *Rhinoclemmys pulcherrima* | Geoemydidae | Testudines | Reptilia | Nicaragua |
|  | KR069082 | *Hemolivia parvula* | *Kinixys zombensis* | Testudinidae | Testudines | Reptilia | South Africa |
|  | KR069083 | *Hemolivia parvula* | *Kinixys zombensis* | Testudinidae | Testudines | Reptilia | South Africa |
| **Reptile, amphibian and mammal hosts** | AB181504 | *Hepatozoon* sp. | *Bandicota indica* | Muridae | Rodentia | Mammalia | Thailand |
|  | AF176837 | *Hepatozoon catesbianae* | bullfrog |  | Anura | Amphibia | NA |
|  | AF297085 | *Hepatozoon boigae* | *Boiga fusca* | Colubridae | Squamata | Reptilia | Australia |
|  | AY252103 | *Hepatozoon* sp. | *Liasis fuscus* | Pythonidae | Squamata | Reptilia | Australia |
|  | AY252108 | *Hepatozoon* sp. | *Varanus scalaris* | Varanidae | Squamata | Reptilia | Australia |
|  | AY252111 | *Hepatozoon* sp. | *Stegonotus cucullatus* | Colubridae | Squamata | Reptilia | Australia |
|  | AY600625 | *Hepatozoon* sp. | *Clethrionomys glareolus* | Cricetidae | Rodentia | Mammalia | Spain |
|  | EF157822 | *Hepatozoon ayorgbor* | *Python regius* | Pythonidae | Squamata | Reptilia | Ghana |
|  | EF222259 | *Hepatozoon* sp. | *Sciurus vulgaris* | Sciuridae | Rodentia | Mammalia | Spain |
|  | FJ719816 | *Hepatozoon* sp. | *Abrothrix sanborni* | Cricetidae | Rodentia | Mammalia | Chile |
|  | FJ719817 | *Hepatozoon* sp. | *Abrothrix olivaceus* | Cricetidae | Rodentia | Mammalia | Chile |
|  | HQ224960 | *Hepatozoon magna* | *Rana esculenta* | Ranidae | Anura | Amphibia | France |
|  | HQ224962 | *Hepatozoon* cf. *clamatae* | *Rana clamitans* | Ranidae | Anura | Amphibia | Canada |
|  | HQ292771 | *Hepatozoon* sp. | *Mabuya wrightii* | Scincidae | Squamata | Reptilia | Seychelles |
|  | HQ292774 | *Hepatozoon* sp. | *Lycognathophis seychellensis* | Colubridae | Squamata | Reptilia | Seychelles |
|  | HQ734787 | *Hepatozoon* sp. | *Tarentola mauritanica* | Phyllodactylidae | Squamata | Reptilia | Algeria |
|  | HQ734790 | *Hepatozoon* sp. | *Ptyodactylus oudrii* | Phyllodactylidae | Squamata | Reptilia | Algeria |
|  | HQ734806 | *Hepatozoon* sp. | *Tarentola mauritanica* | Phyllodactylidae | Squamata | Reptilia | Morocco |
|  | HQ734807 | *Hepatozoon* sp. | *Timon pater* | Lacertidae | Squamata | Reptilia | Morocco |
|  | JQ670908 | *Hepatozoon* sp. | *Aponomma varanense* | Ixodidae | Ixodida | Arachnida | Thailand |
|  | JX244267 | *Hepatozoon* sp. | *Hemorrhois hippocrepis* | Colubridae | Squamata | Reptilia | Spain |
|  | JX531921 | *Hepatozoon* sp. | *Podarcis bocagei* | Lacertidae | Squamata | Reptilia | Portugal |
|  | KC342524 | *Hepatozoon* sp. | *Crotalus durissus* | Viperidae | Squamata | Reptilia | Brazil |
|  | KC342525 | *Hepatozoon* sp. | *Crotalus durissus* | Viperidae | Squamata | Reptilia | Brazil |
|  | KC342526 | *Hepatozoon* sp. | *Crotalus durissus* | Viperidae | Squamata | Reptilia | Brazil |
|  | KC342527 | *Hepatozoon* sp. | *Crotalus durissus* | Viperidae | Squamata | Reptilia | Brazil |
|  | KC696564 | *Hepatozoon* sp. | *Psammophis schokari* | Colubridae | Squamata | Reptilia | Morocco |
|  | KC696566 | *Hepatozoon* sp. | *Psammophis aegyptius* | Colubridae | Squamata | Reptilia | Niger |
|  | KF246565 | *Hepatozoon seychellensis* | *Grandisonia alternans* | Indotyphlidae | Gymnophiona | Amphibia | Seychelles |
|  | KF246566 | *Hepatozoon seychellensis* | *Grandisonia alternans* | Indotyphlidae | Gymnophiona | Amphibia | Seychelles |
|  | KF939620 | *Hepatozoon* sp. | *Elaphe carinata* | Colubridae | Squamata | Reptilia | China |
|  | KF939621 | *Hepatozoon* sp. | *Elaphe carinata* | Colubridae | Squamata | Reptilia | China |
|  | KJ408511 | *Hepatozoon* sp. | *Cerastes cerastes* | Viperidae | Squamata | Reptilia | Mauritania |
|  | KJ408512 | *Hepatozoon* sp. | *Crotaphopeltis hotamboeia* | Colubridae | Squamata | Reptilia | Niger |
|  | KJ408521 | *Hepatozoon* sp. | *Macroprotodon cucullatus* | Colubridae | Squamata | Reptilia | Morocco |
|  | KJ408522 | *Hepatozoon* sp. | *Macroprotodon cucullatus* | Colubridae | Squamata | Reptilia | Morocco |
|  | KJ408523 | *Hepatozoon* sp. | *Macroprotodon cucullatus* | Colubridae | Squamata | Reptilia | Morocco |
|  | KJ408529 | *Hepatozoon* sp. | *Spalerosophis dolichospilus* | Colubridae | Squamata | Reptilia | Morocco |
|  | KJ599676 | *Hepatozoon theileri* | *Amietia quecketti* | Pyxicephalidae | Anura | Amphibia | South Africa |
|  | KM234612 | *Hepatozoon* sp. | *Phyllopezus pollicaris* | Phyllodactylidae | Squamata | Reptilia | Brazil |
|  | KM234613 | *Hepatozoon* sp. | *Phyllopezus pollicaris* | Phyllodactylidae | Squamata | Reptilia | Brazil |
|  | KM234614 | *Hepatozoon* sp. | *Phyllopezus periosus* | Phyllodactylidae | Squamata | Reptilia | Brazil |
|  | KM234615 | *Hepatozoon* sp. | *Hemidactylus mabouia* | Gekkonidae | Squamata | Reptilia | Brazil |
|  | KM234616 | *Hepatozoon* sp. | *Hemidactylus mabouia* | Gekkonidae | Squamata | Reptilia | Brazil |
|  | KM234617 | *Hepatozoon* sp. | *Hemidactylus mabouia* | Gekkonidae | Squamata | Reptilia | Brazil |
|  | KM234618 | *Hepatozoon* sp. | *Hemidactylus mabouia* | Gekkonidae | Squamata | Reptilia | Brazil |
|  | KM234646 | *Hepatozoon* sp. | *Madagascarophis colubrinus* | Lamprophiidae | Squamata | Reptilia | Madagascar |
|  | KM234647 | *Hepatozoon* sp. | *Madagascarophis colubrinus* | Lamprophiidae | Squamata | Reptilia | Madagascar |
|  | KM234648 | *Hepatozoon* sp. | *Ithycyphus oursi* | Lamprophiidae | Squamata | Reptilia | Madagascar |
|  | KM234650 | *Hepatozoon* sp. | *Oplurus* sp. | Opluridae | Squamata | Reptilia | Madagascar |
|  | KU680430 | *Hepatozoon* sp. | *Tarentola boehmei* | Phyllodactylidae | Squamata | Reptilia | Morocco |
|  | KU680441 | *Hepatozoon* sp. | *Tarentola mauritanica* | Phyllodactylidae | Squamata | Reptilia | Morocco |
|  | KU680442 | *Hepatozoon* sp. | *Tarentola fascicularis* | Phyllodactylidae | Squamata | Reptilia | Lybia |
|  | KU680444 | *Hepatozoon* sp. | *Tarentola mauritanica* | Phyllodactylidae | Squamata | Reptilia | Morocco |
|  | KU680450 | *Hepatozoon* sp. | *Tarentola deserti* | Phyllodactylidae | Squamata | Reptilia | Morocco |
|  | KU680460 | *Hepatozoon* sp. | *Tarentola deserti* | Phyllodactylidae | Squamata | Reptilia | Morocco |
|  | KU680464 | *Hepatozoon* sp. | *Tarentola deserti* | Phyllodactylidae | Squamata | Reptilia | Morocco |
|  | KX453573 | *Hepatozoon* sp. | *Sclerophrys arabica* | Bufonidae | Anura | Amphibia | Oman |
|  | KX453581 | *Hepatozoon* sp. | *Sclerophrys arabica* | Bufonidae | Anura | Amphibia | Oman |
|  | KX453597 | *Hepatozoon* sp. | *Hemidactylus alkiyumii* | Gekkonidae | Squamata | Reptilia | Oman |
|  | KX453599 | *Hepatozoon* sp. | *Hemidactylus lemurinus* | Gekkonidae | Squamata | Reptilia | Oman |
|  | KX453613 | *Hepatozoon* sp. | *Pristurus rupestris* | Sphaerodactylidae | Squamata | Reptilia | Oman |
|  | KX453628 | *Hepatozoon* sp. | *Asaccus platyrhynchus* | Phyllodactylidae | Squamata | Reptilia | Oman |
|  | KX453637 | *Hepatozoon* sp. | *Cerastes gasperettii* | Viperidae | Squamata | Reptilia | Oman |
|  | KX453640 | *Hepatozoon* sp. | *Echis omanensis* | Viperidae | Squamata | Reptilia | Oman |
| **Carnivore hosts** | AF176836 | *Hepatozoon americanum* | *Amblyomma maculatum* | Ixodidae | Ixodida | Arachnida | NA |
|  | AY461375 | *Hepatozoon canis* | *Cerdocyon thous* | Canidae | Carnivora | Mammalia | Brazil |
|  | AY628681 | *Hepatozoon felis* | *Felis catus* | Felidae | Carnivora | Mammalia | Spain |
|  | AY731062 | *Hepatozoon canis* | *Vulpes vulpes* | Canidae | Carnivora | Mammalia | Spain |
|  | DQ111754 | *Hepatozoon canis* | *Canis lupus* | Canidae | Carnivora | Mammalia | Sudan |
|  | EF222257 | *Hepatozoon* sp. | *Martes martes* | Mustelidae | Carnivora | Mammalia | Spain |
|  | HQ829446 | *Hepatozoon felis* | *Panthera tigris* | Felidae | Carnivora | Mammalia | India |
| **Lacertid, snake, skink and varanid hosts** | EU908289 | *Hepatozoon* sp. | *Lacerta agilis* | Lacertidae | Squamata | Reptilia | Poland |
|  | HQ734791 | *Hepatozoon* sp. | *Scelarcis perspicillata* | Lacertidae | Squamata | Reptilia | Morocco |
|  | HQ734792 | *Hepatozoon* sp. | *Podarcis vaucheri* | Lacertidae | Squamata | Reptilia | Morocco |
|  | HQ734793 | *Hepatozoon* sp. | *Podarcis vaucheri* | Lacertidae | Squamata | Reptilia | Morocco |
|  | HQ734795 | *Hepatozoon* sp. | *Podarcis vaucheri* | Lacertidae | Squamata | Reptilia | Morocco |
|  | HQ734796 | *Hepatozoon* sp. | *Eumeces algeriensis* | Scincidae | Squamata | Reptilia | Morocco |
|  | HQ734798 | *Hepatozoon* sp. | *Atlantolacerta andreanskyi* | Lacertidae | Squamata | Reptilia | Morocco |
|  | HQ734799 | *Hepatozoon* sp. | *Timon pater* | Lacertidae | Squamata | Reptilia | Morocco |
|  | JX244268 | *Hepatozoon* sp. | *Hemorrhois hippocrepis* | Colubridae | Squamata | Reptilia | Morocco |
|  | JX531910 | *Hepatozoon* sp. | *Podarcis hispanica* | Lacertidae | Squamata | Reptilia | Spain |
|  | JX531917 | *Hepatozoon* sp. | *Podarcis hispanica* | Lacertidae | Squamata | Reptilia | Spain |
|  | JX531920 | *Hepatozoon* sp. | *Podarcis lilfordi* | Lacertidae | Squamata | Reptilia | Spain |
|  | JX531941 | *Hepatozoon* sp. | *Algyroides marchi* | Lacertidae | Squamata | Reptilia | Portugal |
|  | KJ189397 | *Hepatozoon* sp. | *Podarcis bocagei* | Lacertidae | Squamata | Reptilia | Portugal |
|  | KJ189404 | *Hepatozoon* sp. | *Podarcis hispanica* | Lacertidae | Squamata | Reptilia | Portugal |
|  | KJ189425 | *Hepatozoon* sp. | *Podarcis hispanica* | Lacertidae | Squamata | Reptilia | Portugal |
|  | KJ408524 | *Hepatozoon* sp. | *Malpolon moilensis* | Colubridae | Squamata | Reptilia | Morocco |
|  | KJ461939 | *Karyolysus* sp. | *Podarcis muralis* | Lacertidae | Squamata | Reptilia | Slovakia |
|  | KJ461940 | *Karyolysus* sp. | *Lacerta viridis* | Lacertidae | Squamata | Reptilia | Poland |
|  | KJ461941 | *Karyolysus* sp. | *Ixodes riciinus* | Ixodidae | Ixodida | Arachnida | Hungary |
|  | KJ461944 | *Karyolysus* sp. | *Ophionyssus* sp. | Macronyssidae | Mesostigmata | Arachnida | Hungary |
|  | KU680457 | *Hepatozoon* sp. | *Tarentola angustimentalis* | Phyllodactylidae | Squamata | Reptilia | Spain |
|  | KX011040 | *Karyolysus paradoxa* | *Varanus albigularis* | Varanidae | Squamata | Reptilia | South Africa |
|  | KX453642 | *Hepatozoon* sp. | *Echis carinatus* | Viperidae | Squamata | Reptilia | Oman |
| **Gecko hosts** | KU680434 | *Hepatozoon* sp. | *Tarentola ephippiata* | Phyllodactylidae | Squamata | Reptilia | Morocco |
|  | KU680435 | *Hepatozoon* sp. | *Tarentola mauritanica* | Phyllodactylidae | Squamata | Reptilia | Morocco |
|  | KU680438 | *Hepatozoon* sp. | *Tarentola mauritanica* | Phyllodactylidae | Squamata | Reptilia | Morocco |
|  | KU680445 | *Hepatozoon* sp. | *Tarentola mauritanica* | Phyllodactylidae | Squamata | Reptilia | Morocco |
|  | KU680448 | *Hepatozoon* sp. | *Tarentola mauritanica* | Phyllodactylidae | Squamata | Reptilia | Algeria |
|  | KU680455 | *Hepatozoon* sp. | *Tarentola mauritanica* | Phyllodactylidae | Squamata | Reptilia | Morocco |
|  | KU680462 | *Hepatozoon* sp. | *Tarentola mauritanica* | Phyllodactylidae | Squamata | Reptilia | Morocco |
|  | KU680463 | *Hepatozoon* sp. | *Tarentola mauritanica* | Phyllodactylidae | Squamata | Reptilia | Morocco |
|  | KX453590 | *Hepatozoon* sp. | *Hemidactylus hajarensis* | Gekkonidae | Squamata | Reptilia | Oman |
|  | KX453595 | *Hepatozoon* sp. | *Asaccus platyrhynchus* | Phyllodactylidae | Squamata | Reptilia | Oman |
|  | KX453600 | *Hepatozoon* sp. | *Hemidactylus hajarensis* | Gekkonidae | Squamata | Reptilia | Oman |
|  | KX453647 | *Hepatozoon* sp. | *Hemidactylus hajarensis* | Gekkonidae | Squamata | Reptilia | Oman |
