# Supplementary material for: Along for the ride or missing it altogether: exploring the host specificity and diversity of haemogregarines in the Canary Islands
Source: Parasit Vectors. 2018 Mar 19;11:190. doi: 10.1186/s13071-018-2760-5 (PMC5859493; doi:10.1186/s13071-018-2760-5)
Supplement: Supplementary file 4 — Table S4 Statistical summary of the variation of haemogregarine prevalence and parasitaemia from Canarian lizards. (DOCX 18 kb) [file 13071_2018_2760_MOESM4_ESM.docx]

**Additional file 4: Table S4.** Statistical summary of the variation of haemogregarine prevalence and parasitaemia from Canarian lizards.

| **INFECTION PER HOST GENUS AND ISLAND** | | | | | |
| --- | --- | --- | --- | --- | --- |
|  |  | Prevalence | Parasitaemia | | |
|  |  |  | mean (±sd) | min−max | n |
| Overall total | | 36.5 | 1.06 (±3.02) | 0.02−29.48 | 285 |
| **Host genus** | Island |  |  |  |  |
| ***Gallotia*** | Total | 69.7 | 1.07 (±3.07) | 0.02−29.48 | 272 |
|  | El Hierro | 80.0 | 1.03 (±3.80) | 0.02−24.64 | 44 |
|  | La Palma | 81.1 | 0.69 (±0.87) | 0.02−3.96 | 42 |
|  | La Gomera | 52.2 | 0.16 (±0.18) | 0.02−0.84 | 24 |
|  | Tenerife | 84.4 | 0.55 (±0.79) | 0.02−4.32 | 54 |
|  | Gran Canaria | 18.2 | 0.31 (±0.51) | 0.02−1.40 | 7 |
|  | Fuerteventura | 71.2 | 0.58 (±0.81) | 0.02−4.40 | 46 |
|  | Lanzarote | 81.6 | 2.82 (±5.48) | 0.04−29.48 | 55 |
|  |  |  |  |  |  |
| ***Tarentola*** | Total | 4.9 | 1.07 (±1.52) | 0.02−4.36 | 8 |
|  | La Palma | 21.2 | 1.72(±1.92) | 0.28−4.36 | 4 |
|  | La Gomera | 17.2 | 0.55(±0.91) | 0.02−1.60 | 3 |
|  | Tenerife | 2.3 | 0.04 | - | 1 |
|  |  |  |  |  |  |
| ***Chalcides*** | Total | 3.3 | 0.05 (±0.06) | 0.02−0.16 | 5 |
|  | El Hierro | 2.8 | 0.02 | - | 1 |
|  | La Gomera | 6.3 | 0.02 | - | 1 |
|  | Tenerife | 13.0 | 0.07(±0.08) | 0.02−1.60 | 3 |
|  |  |  |  |  |  |
| **INFECTION PER HAEMOGREGARINE HAPLOTYPE** | | | | | |
|  |  |  | Parasitaemia | | |
| **Haplotype** | Host genus |  | mean (±sd) | min−max | n |
| **A** | *Gallotia* | - | 1.15 (±1.52) | 0.04−7.28 | 27 |
| **B** | *Gallotia* | - | 1.38 (±3.58) | 0.02−24.64 | 48 |
|  | *Tarentola* | - | 0.04 | - | 1 |
|  | *Chalcides* | - | 0.02 | - | 1 |
| **C** | *Gallotia* | - | 0.31 (±0.51) | 0.02−1.40 | 7 |
| **D** | *Gallotia* | - | 1.16 (±0.15) | 0.02−0.56 | 31 |
| **E** | *Gallotia* | - | 1.33 (±0.16) | 0.04−0.32 | 3 |
| **F** | *Chalcides* | - | 0.07 (±0.08) | 0.02−0.16 | 3 |
| **T** | *Tarentola* | - | 1.42 (±1.63) | 0.04−4.36 | 6 |

Prevalence and parasitaemia (mean, range and sample size) for the three host genera, in total and per island, and parasitaemia for the nine discovered haemogregarine haplotypes (only genotyped samples without mixed-infected samples were considered for these estimates). The values shown for prevalence are the percentage of samples infected with haemogregarines, and for parasitaemia are the percentage of haemogregarine-infected cells per 2,500 erythrocytes.
