# Supplementary figures and images for: Along for the ride or missing it altogether: exploring the host specificity and diversity of haemogregarines in the Canary Islands
Source: Parasit Vectors. 2018 Mar 19;11:190. doi: 10.1186/s13071-018-2760-5 (PMC5859493; doi:10.1186/s13071-018-2760-5)

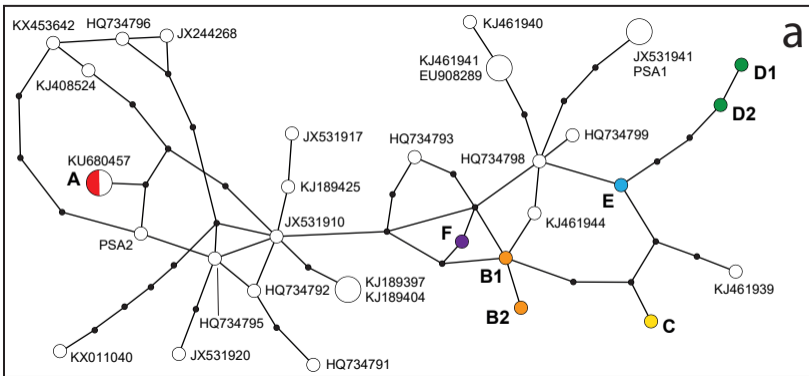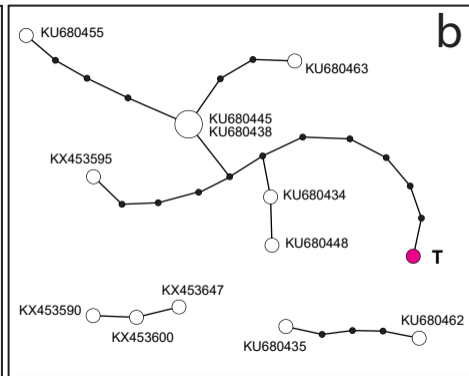

Supplement: Supplementary file 5 — Figure S1. Networks of the haemogregarine haplotypes infecting Gallotia, Chalcides (a) and Tarentola lizards (b). Represented is the network estimations performed with TCS. Black nodes represent mutations, while coloured ones correspond to the sequences of Canarian haemogregarines (colours match the ones used in Fig. 3), and the white ones correspond to the remaining samples. The network of a includes the samples of the haemogregarine clade from lacertid, snake, skink and varanid hosts (as identified in Additional file 4: Table S4) and b includes the samples of the clade from gecko hosts. (PDF 911 kb) [file 13071_2018_2760_MOESM5_ESM.pdf]
